# Low-Grade Inflammation Is Not Present in Former Obese Males but Adipose Tissue Macrophage Infiltration Persists

**DOI:** 10.3390/biomedicines8050123

**Published:** 2020-05-14

**Authors:** Ignacio Ara, Pernille Auerbach, Steen Larsen, Esmeralda Mata, Bente Stallknecht, Thorkil Ploug, Clara Prats, Jørn W. Helge

**Affiliations:** 1 Growth, Exercise, Nutrition and Development—GENUD Toledo Research Group, Universidad de Castilla-La Mancha, 45071 Toledo, Spain; Ignacio.Ara@uclm.es (I.A.); esmeralda.mata@empoliclinica.com (E.M.); 2 Biomedical Research Networking Center on Frailty and Healthy Aging (CIBERFES), 28029 Madrid, Spain; 3 Xlab, Center for Healthy Aging, Department of Biomedical Sciences, University of Copenhagen, 2200N Copenhagen, Denmark; stelar@sund.ku.dk (S.L.); cprats@sund.ku.dk (C.P.); 4 Department of Biomedical Sciences, University of Copenhagen, 2200N Copenhagen, Denmark; pernille.auerbach@gmail.com (P.A.); bstall@Sund.ku.dk (B.S.); tploug@sund.ku.dk (T.P.); 5 Clinical Research Centre, Medical University of Bialystok, 15-089 Bialystok, Poland

**Keywords:** obesity, abdominal, gluteal, cytokines, weight loss

## Abstract

Macrophage infiltration in two subcutaneous adipose tissue depots and systemic low-grade inflammation were studied in post-obese (PO), obese (O), and control (C) subjects. Young males were recruited into PO: (*n* = 10, weight-loss avg. 26%, BMI: 26.6 ± 0.7, mean ±SEM kg/m^2^), O: (*n* = 10, BMI: 33.8 ± 1.0kg/m^2^) and C: (*n* = 10, BMI: 26.6 ± 0.6 kg/m^2^). PO and C were matched by BMI. Blood and abdominal and gluteal subcutaneous adipose tissue were obtained in the overnight fasted state. Plasma concentrations of IL-6 and CRP were higher (*p* < 0.05) in O than in PO and C, TNF-α was higher (*p* < 0.05) only in O compared to PO and IL-18 was similar between groups. The number of CD68^+^ macrophages was higher (*p* < 0.05) in the gluteal than the abdominal depot, and higher (*p* < 0.05) in O and PO compared to C in both depots. The content of CD163^+^ macrophages was similar between depots but was higher (*p* < 0.05) in PO compared to C and O in the gluteal depot. In post obese men with a long-term sustained weight loss, systemic low-grade inflammation was similar to non-obese controls despite a higher subcutaneous adipose tissue CD68^+^ macrophage content. Interestingly, the anti-inflammatory CD163^+^ macrophage adipose tissue content was consistently higher in post obese than obese and controls.

## 1. Introduction

Obesity is associated with a chronic low-grade inflammatory state that predisposes to insulin resistance, type 2 diabetes, and cardiovascular disease [1,2,3]. Chronic low-grade inflammation is characterized by elevated systemic levels of inflammatory markers such as C-reactive protein (CRP), interleukin-6 (IL-6), interleukin-18 (IL-18), and tumour necrosis factor-α (TNF-α) [4,5]. The obesity-associated low-grade inflammation is associated with a higher content of macrophages in the adipose tissue of obese compared to lean subjects [6,7,8,9]. Studies have shown that, depending on the stimuli, macrophages can transform into specialized subtypes with different functional properties [10,11]. Evidence from in vitro cell culture studies suggests that polarization into subtypes M1 and M2 may determine macrophage function [12]. M1 or “classically activated” macrophages produce pro-inflammatory cytokines such as IL-6 and TNF-α. In contrast, M2 or “alternatively activated” macrophages produce high levels of anti-inflammatory cytokines such as IL-10 and interleukin-1 receptor antagonist (IL-1ra) [10,11,13]. In the shorter term (1 to 6 months), weight loss studies in obese people, the total content of macrophages, and M1 macrophages have been shown to decrease in adipose tissue [14,15,16]. The picture is less clear for the anti-inflammatory M2 macrophages where both an increase and a decrease have been reported after weight loss [15,16,17]. In a long-term study in which gastric bypass patients were followed for 18 months, the overall subcutaneous adipose tissue macrophage content remained unaltered despite a major weight loss [18]. Interestingly, plasma IL-6, IL-18, and TNFα concentrations remained unchanged after gastric bypass, but the CRP concentration had decreased 18 months post-surgery [18]. Other studies have demonstrated a reduction of systemic low-grade inflammation after weight loss [19,20]. Overall, it remains somewhat unclear whether adipose tissue macrophage content and polarization are normalized after long-term weight loss.

Human adipose tissue is a heterogeneous tissue that is distributed across a number of depots in the body. There has been persistent and on-going debate about the specific function and importance of differently located subcutaneous adipose tissue depots [21]. Evans and colleagues demonstrated a higher expression of inflammatory markers in gluteal compared to abdominal subcutaneous adipose tissue in both white and black women [22]. It is at present not clear whether macrophage infiltration varies across subcutaneous adipose tissue depots and to what extent this is affected by obesity and subsequent reductions in fat content.

Therefore, our aim was to investigate the relationship between systemic low-grade inflammation and macrophage infiltration in two subcutaneous adipose tissue depots in post obese, obese, and control male subjects.

## 2. Materials and Methods

### 2.1. Subjects

Thirty young male subjects participated in the study. Subjects were fully informed of the nature and the possible risks associated with the study before giving written consent to participate. The study was approved by the Copenhagen Ethics Committee (KF 01 304792, Approved 22 May 2006) and the experiments conformed to The Declaration of Helsinki. We have published a separate paper with a different scientific focus using the same subjects that are included in this paper [23].

### 2.2. Experimental Protocol

The experimental design and recruitment have been described in detail in a prior paper [23]. In brief, the subjects were recruited through advertisements in sports clubs, voluntary organizations, local supermarkets, and dorms as well as newspaper advertisements, and were recruited into three groups: Post obese, Obese, and Control. The inclusion criteria for the Post obese were: (1) BMI lower than 30 kg/m^2^, (2) weight loss from an obese condition (BMI higher than 30 kg/m^2^) through lifestyle-based, non-pharmacological and non-surgical therapies of at least 10% of body weight (on average the weight loss was 26% (range 15–37%), and (3) weight stability at the time of the study (±2 kg for at least one month prior to the beginning of the experiments). The weight loss of the Post obese subjects had on average occurred 6 ± 1 (mean ± SEM) year’s earlier (range 1–10 years). For the Obese subjects, a BMI higher than 30 kg/m^2^ was required. Subjects from all three groups were healthy and not taking any medication. Intentionally, the three groups were matched by age and the Control and Post obese also by BMI.

Subjects reported to the laboratory on three separate days. Subjects were instructed not to engage in vigorous physical activity on the day before each test day and to consume their normal diet avoiding excesses in alcohol and tobacco consumption. On the experimental days, subjects came overnight fasted and on the first day a venous blood sample was obtained after 15 min rest in a supine position. After this, body composition was determined by dual-energy X-ray absorptiometry scanning using a Lunar Prodigy Advance bone densitometer (Lunar Corporation, Madison, WI, USA). Finally, a graded incremental exercise protocol was used to establish VO_2peak_ on a standard bicycle ergometer (Ergometrics 800, Jaeger, Würzburg, Germany) measured with an on-line system (Oxycon Pro; Jaeger, Würzburg, Germany).

On the two last experimental days, subjects were placed in a supine resting position for 30 min. Subsequently, a biopsy was obtained from either subcutaneous abdominal or gluteal adipose tissue depots applying the Bergström needle biopsy technique [24]. In brief, the skin of the periumbilical or gluteal area was anesthetized with lidocaine (10 mg/mL) and a small incision was made. Approximately, 300 mg of adipose tissue was removed under sterile conditions.

The full experimental protocol applied on day 2 and 3 has previously been described in detail [23].

### 2.3. Analytical Procedures

Blood was sampled and transferred into tubes containing 0.3M ethylene-diamine-tetra-acetic acid (EDTA) (10 mg/mL blood) and immediately centrifuged for 10 min at 4 °C and stored at −80 °C until later analysis. Plasma IL-6, TNF-α, and IL-18 were measured with high-sensitivity (HS) ELISA kits from R&D Systems (Minneapolis, MN, USA) and plasma CRP using a conventional HS assay on a Hitachi (Roche cat. No. 11972855 216, Roche Daignostics, Indianapolis, IN, USA). Analysis of plasma insulin and glucose has been described previously [23]. Insulin sensitivity was estimated through calculation of the Quicki index [25].

The description of the adipose tissue fixation and analysis procedure can be found in full detail in a former publication [15]. In brief, adipose tissue biopsies were fixed in 2% Zamboni fixative, embedded in paraffin and sectioned (5 μm thick) on a microtome (Reichter, Munich, Germany). In order to visualize the general morphology of subcutaneous adipose tissue, sections were stained with Mayer’s haematoxylin (S3309, Dako) and 2% eosin (1345, Merck, NJ, USA).

The immunostaining of macrophages were performed with primary antibody against CD68 (diluted 1:200, Abcam, Cambridge, UK) and CD163 (diluted 1:200, Visionbiosystems Novocastra, Newcastle, UK) and detected using Dako’s REAL Envision Detection system (K5007, Dako, Copenhagen, Denmark) and 3,3′-diaminobenzidine as a chromogen. Sections with no primary antibodies were included as negative controls. Image acquisition of the stained sections was performed with a Panoramic slide scanner (Zeiss, Oberkochen, Germany). The quantification was in essence performed as introduced by Cancello et al. [14] and further described by Auerbach et al. and Kristensen et al. [15,18]. CD163^+^ and CD68^+^ cells representing different subsets of adipose tissue macrophages (anti-inflammatory (M2) and a marker for macrophages not committed to the M2 lineage, respectively) were counted along with adipocytes by an observer blinded for slide identity in eight randomly chosen areas (magnified × 20) within each slide, representing overall for both depots an area of 272 ± 6 adipocytes and excluding macrophages located in abundant stroma-vascular areas. The total number of CD68^+^ or CD163^+^ macrophages in each slide was expressed as a percentage of the total number of adipocytes counted.

**Figure 1:**
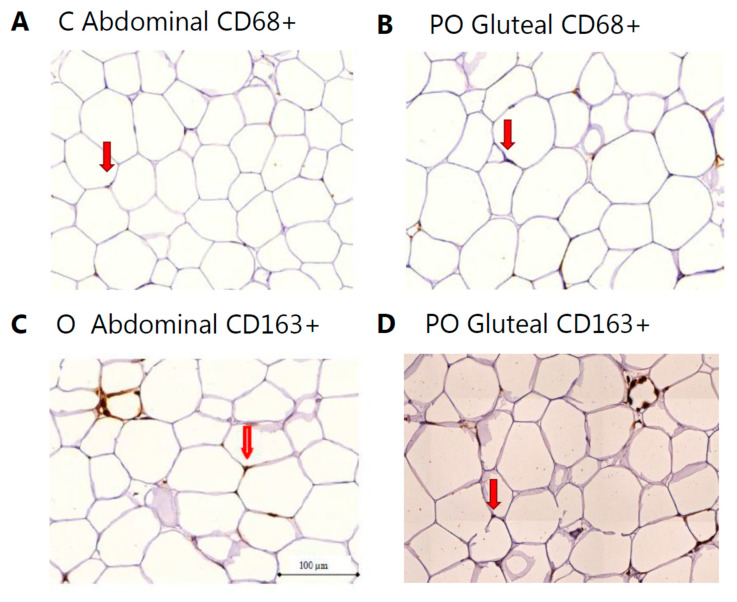
Representative images of macrophage infiltration in different subjects from PO, O, and C of CD68^+^ (**a**,**b**) and CD163^+^ (**c**,**d**) cells in subcutaneous abdominal and gluteal adipose tissue depots, respectively. The red arrows point to a positive staining of the corresponding marker.

The cross-sectional adipocyte area was determined using Adobe Photoshop CS4 Extended Version 11.0.1 (Adobe Systems Incorporated, San Jose, CA, USA). Areas were determined by color thresholding (Figure 1). Background variations due to uneven illumination were compensated with the “high pass filter”.

### 2.4. Statistics

Results are given as means ± SEM, if not otherwise stated. One-way and two-way analyses of variance (ANOVA) with repeated measures for the location factor were performed. When appropriate, the Holm–Sidak post hoc test was applied to discern differences at the group or tissue level. Pearson bivariate correlations were applied to identify the relationship between mean adipocyte area, body fat percentage, and the number of macrophage markers. In all cases, two-tailed testing was applied and *p* < 0.05 was used as the level of significance. The statistical analysis was performed using Sigma Stat 3.1 (Sigmastat, SPSS Inc., Erkrath, Germany).

## 3. Results

Age, height, lean body mass, and maximal oxygen uptake (VO_2peak_) were similar in the three groups (Table 1). Body weight, body fat, and BMI were higher (*p* < 0.05) in Obese compared to the two other groups but similar between Post obese and Control (Table 1). The quantitative insulin sensitivity check index (QUICKI) was lower (*p* < 0.05) in the Obese than in the other groups (Table 1). A more detailed characteristic of the subjects including data on plasma insulin and glucose is available in a prior publication [23].

Plasma IL-6 and CRP concentrations were significantly higher in Obese compared to Post obese and Control, whereas TNF-α was only significantly higher in Obese compared to Post obese (Table 2). The plasma IL-18 concentration was not significantly different between the three groups although a tendency (*p* = 0.07) towards higher values in Obese compared to the other groups was evident (Table 2). Comparable levels of plasma IL-6, TNF-α, IL-18, and CRP were present in Post obese and Control.

For both adipose tissue depots, Obese tended (*p* = 0.06) to have larger adipocyte areas compared to the two other groups (Table 2). The mean adipocyte area was similar in abdominal and gluteal depots, and adipocyte areas from the two depots were positively correlated (R = 0.46, *p* < 0.05, *n* = 23). The percentage of body fat correlated only with gluteal adipocyte area (R = 0.54, *p* < 0.01, *n* = 24). Due to technical limitations, adipocyte area could not be determined in all samples. No significant correlations were found between the content of macrophage markers (CD68^+^ and CD163^+^) and the adipocyte area, neither in the abdominal nor in the gluteal adipose tissue depot.

**Figure 2:**
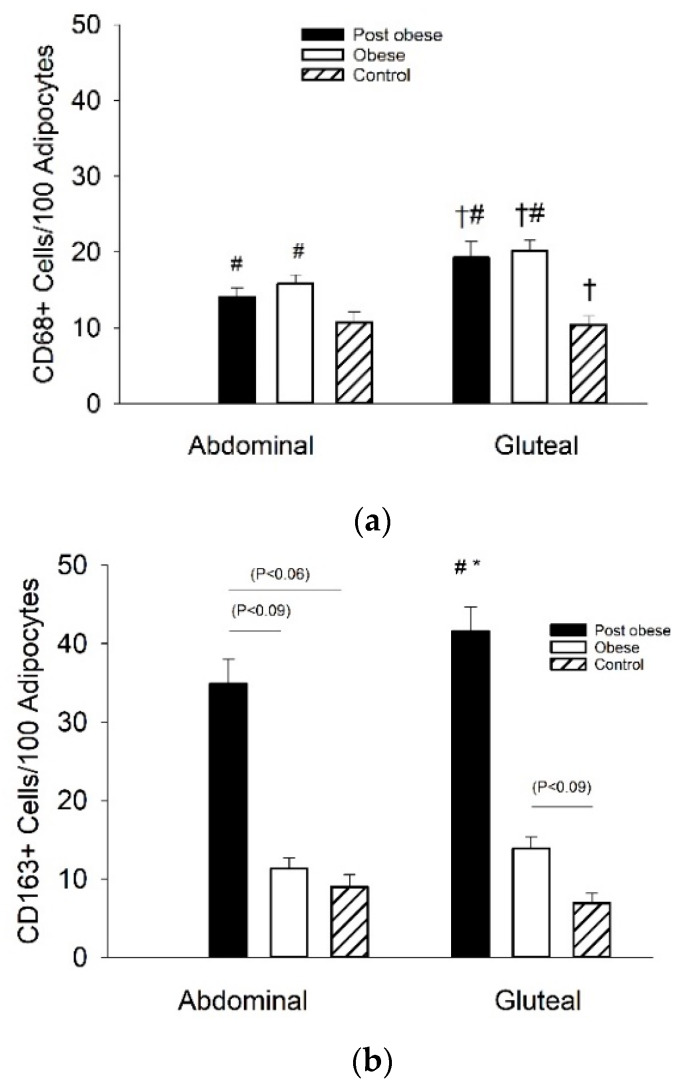
Adipocyte macrophage markers CD68^+^ (**a**) and CD163^+^ (**b**) in subcutaneous abdominal (*n* = 7, *n* = 8, *n* = 9 and *n* = 7, *n* = 8, and *n* = 9, respectively) and gluteal (*n* = 8, *n* = 9, *n* = 10 and *n* = 7, *n* = 8, *n* = 10, respectively) depots from Post obese, Obese and Control male subjects, respectively. Data are mean ± SEM. *: (*p* < 0.05) vs. Obese; #: (*p* < 0.05) vs. Control; †: (*p* < 0.05) Abdominal vs. Gluteal.

The content of CD68^+^ cells was higher (*p* < 0.05) in the gluteal compared to the abdominal depot (Figure 2a). In both adipose tissue depots, Post obese and Obese had a higher (*p* < 0.05) content of CD68^+^ cells than Controls.

A significant group—depot interaction (*p* < 0.05) was apparent for the content of CD163^+^ cells (Figure 2b). In the gluteal depot, the Post obese had higher (*p* < 0.05) content of CD163^+^ cells than the two other groups (Figure 2b). There was also a tendency (*p* = 0.09) towards a higher content of CD163^+^ cells in Obese compared to Controls in the gluteal depot. In the abdominal depot, there was a trend towards a higher content of CD163^+^ cells in Post obese vs. Control (*p* < 0.06) and Obese (*p* < 0.09). The content of CD163^+^ cells did not vary between the two adipose tissue depots. The CD68^+^ and CD163^+^ stain densities positively correlated in the abdominal (R = 0.59, *p* < 0.01, *n* = 27) and the gluteal (R = 0.67, *p* < 0.01, *n* = 27) adipose tissue depots.

## 4. Discussion

A novel finding in this study was that the content of CD68^+^ macrophages was similar between Post obese and Obese in both abdominal and gluteal adipose tissue and that the CD68^+^ macrophage content was higher than in matched Controls. Despite the difference in adipose tissue macrophages, systemic low-grade inflammation was similar in Post obese and Control and notably lower than in Obese. Another novel observation was the markedly higher adipose tissue anti-inflammatory CD163^+^ content in Post obese compared to both Obese and Control, which may have influenced the systemic low-grade inflammation.

In this study, we took advantage of a cross sectional study design and recruited former obese subjects that had succeeded in sustaining a marked weight loss over several years. In line with prior findings, where diet or diet and exercise [19,26] resulted in a weight loss-induced decrease in systemic low-grade inflammation, we observed that levels of systemic low-grade inflammatory markers in Post obese were similar to age- and BMI- matched Controls and lower than in age-matched Obese. However, this contrasts with the observation by Kristensen et al. where systemic low grade inflammation (IL-6, IL-18, and TNFα concentrations) was not changed 18 months after gastric bypass-induced weight loss [18]. Duration (18 months vs. average 6 years) and mode (gastric bypass vs. lifestyle change) of weight loss may explain the difference.

In the present study, the CD68^+^ macrophage infiltration was higher in Post obese compared to Control in both adipose tissue depots, which was somewhat unexpected given the normalization of the low-grade inflammation. Interestingly, there is a discrepancy in the literature, where some studies find a decreased macrophage infiltration in adipose tissue from obese [16,19] and morbidly obese [14] with weight loss, but other studies do not find changes both with significant weight loss (>10%) [18,27] or with a limited weight loss [28,29]. Forsythe and colleagues suggested that a certain level of weight loss (>10%) is needed to alter low-grade inflammation and adipose tissue macrophage infiltration [30]. More recently, Magkos and colleagues speculated that major weight loss may induce a biphasic response in adipose tissue inflammation with an initial increase followed by a decrease [31]. However, the results of both the current study and the study by Kristensen et al. [18] do not support this speculation. Albeit, there is still a lack of longitudinal studies, where this is studied in subjects with normal BMI after long-term weight loss.

In the present study, the identification and quantification of macrophages in adipose tissue were based on antibodies against CD68^+^ and CD163^+^ markers that are both highly expressed by human monocytes and tissue macrophages [11,32]. These antibodies are applied as a none committed macrophage marker (CD68) or a marker of macrophages committed to the M2 line (CD163), and have been used in previous studies for the quantification of adipose tissue macrophages in humans [14,15,18]. There is evidence that macrophages change from M2 to an M1 polarization in obese adipose tissue, leading to a more inflamed tissue and subsequently a higher release of pro-inflammatory cytokines [11,33,34]. Interestingly, we observed a two-fold higher content of CD163^+^ (M2) macrophages in adipose tissue of former obese subjects when compared to both obese and BMI-matched controls. In support for this finding, a higher subcutaneous adipose tissue M2 macrophage content was reported both after 12 weeks training-induced weight loss [15] and 3 months after gastric bypass [17]. We have previously reported a higher plasma adiponectin concentration in the Post obese compared to the two other groups [23], and since adiponectin promotes a shift in macrophage polarization towards the M2 state [35,36], one could speculate that higher adiponectin may therefore provide a mechanism to activate commitment of macrophages towards an anti-inflammatory M2 (CD163^+^) phenotype in Post obese. Overall, this suggests that a permanently maintained substantial weight loss leads to a higher content of M2 state macrophages through a change in polarization and/or a recruitment of new macrophages, rather than an attenuation of the number of adipose tissue macrophages.

In the present study, CD68^+^ macrophage infiltration was higher in the gluteal compared to the abdominal subcutaneous adipose tissue depot. This effect was primarily due to CD68^+^ macrophage content being approximately 25% higher for Post obese and Obese in the gluteal compared to the abdominal depot, whereas the number of CD68^+^ macrophages was similar in gluteal and abdominal adipose tissue of the Control subjects (Figure 2A). In line with this observation, Evans and colleagues observed that in both white and black women the gluteal subcutaneous adipose tissue had lower expression of adiponectin, but higher expression of inflammatory cytokines, macrophage markers and leptin than abdominal subcutaneous adipose tissue [22]. You and colleagues extended this finding and demonstrated improved adiponectin release only in abdominal but not gluteal adipose tissue after 20 weeks of training- and diet-induced weight loss in 42 obese women [37]. However, two other studies, albeit with a limited number of subjects, could not detect a difference in adiponectin gene expression between subcutaneous abdominal and gluteal adipose tissue depots [38,39].

The present study applied a cross-sectional design and thus cannot exclude that the observed differences are due to inherent genetic traits rather than fluctuations in body weight. We applied two macrophage markers, where CD68^+^ is somewhat non-specific and CD163^+^ very specific, and although this difference in specificity is not ideal, we do think that the comparisons between tissues and across groups are valid. Furthermore, it is important to note that the BMI of the Control group is matched with the Post obese and is thus not within the BMI range considered normal, since we were unable to recruit all the Post obese subjects with a normal BMI. However, a strength of this study is the very fine matching of the subjects.

## 5. Conclusions

In conclusion, obesity-associated systemic low-grade inflammation is reversible and returns to levels comparable to age- and BMI-matched controls after a long-term sustained weight loss. Moreover, the present study indicates that normalization of low-grade inflammation with a long-term maintained weight loss is not concurrent with a decrease in adipose tissue macrophage content, but paralleled by a change in macrophage polarization and an increase in the number of resident anti-inflammatory macrophages.

## Figures and Tables

**Table 1:** Characteristics of Post obese, Obese, and Control male subjects.

	POST OBESE (*n* = 10)			OBESE (*n* = 10)			CONTROL (*n* = 10)		
Age (years)	31.5	±	1.6	30.4	±	2.3	31.2	±	1.5
Height (cm)	184.0	±	1.7	183.9	±	2.6	184.3	±	3.0
Weight (kg)	90.2	±	3.1 *	115.0	±	5.4	90.9	±	4.2 *
BMI (kg/m^2^)	26.6	±	0.7 *	33.8	±	1.0	26.6	±	0.6 *
Body fat (%)	22.8	±	1.8 *	34.9	±	1.6	24.7	±	1.8 *
LBM (kg)	66.8	±	1.5	69.2	±	3.2	65.5	±	1.9
VO_2peak_ (L/min)	3.7	±	0.1	3.4	±	0.2	3.6	±	0.1
QUICKI	0.38	±	0.01 *	0.33	±	0.01	0.37	±	0.01 *

Data are mean ± SEM. Abbreviations; Body mass index (BMI), lean body mass (LBM); peak oxygen consumption (VO_2peak_), Quantitative insulin sensitivity check index (QUICKI). *: (*p* < 0.05) vs. Obese.

**Table 2:** Systemic low-grade inflammation and adipocyte area in Post obese, Obese, and Control male subjects.

	POST OBESE (*n* = 9)			OBESE (*n* = 10)			CONTROL (*n* = 10)		
Plasma								
TNF-α (pg/mL)	1.28	±	0.16 *	1.79	±	0.08	1.39	±	0.13
IL-18 (pg/mL)	327	±	34	469	±	50	389	±	40
IL-6 (pg/mL)	0.98	±	0.32 *	1.66	±	0.24	0.92	±	0.22 *
CRP (mg/L)	0.76	±	0.17 *	3.84	±	1.04	1.92	±	1.01 *
Adipocyte Area									
Abdominal depot (10^3^ µm^2^)	10.2	±	0.7	12.8	±	0.7	11.2	±	0.7
Gluteal depot (10^3^ µm^2^)	10.0	±	0.9	12.1	±	0.8	10.5	±	0.7

Data are mean ± SEM. *: (*p* < 0.05) vs. Obese.
